# Association Between Region of Birth and Advance Care Planning Documentation Among Older Australian Migrant Communities: A Multicenter Audit Study

**DOI:** 10.1093/geronb/gbaa127

**Published:** 2020-08-17

**Authors:** Craig Sinclair, Marcus Sellars, Kimberly Buck, Karen M Detering, Ben P White, Linda Nolte

**Affiliations:** 1 School of Psychology, University of New South Wales, Sydney, Australia; 2 Centre of Excellence in Population Ageing Research, University of New South Wales, Sydney, Australia; 3 Advance Care Planning Australia, Austin Health, Melbourne, Australia; 4 Australian Centre for Health Research Law, Faculty of Law, Queensland University of Technology, Brisbane, Australia; 5 Faculty of Medicine, Dentistry and Health Sciences, University of Melbourne, Parkville, Australia

**Keywords:** Advance care directive, Autonomy, Cultural and linguistic diversity, End-of-life care

## Abstract

**Objectives:**

This study explored associations between birth region, sociodemographic predictors, and advance care planning (ACP) uptake.

**Methods:**

A prospective, multicenter, cross-sectional audit study of 100 sites across 8 Australian jurisdictions. ACP documentation was audited in the health records of people aged 65 years or older accessing general practice (GP), hospital, and long-term care facility (LTCF) settings. Advance care directives (ACDs) completed by the person (“person completed ACDs”) and ACP documents completed by a health professional or other person (“health professional or someone else ACP”) were counted. Hierarchical multilevel logistic regression assessed associations with birth region.

**Results:**

From 4,187 audited records, 30.0% (1,152/3,839) were born outside Australia. “Person completed ACDs” were less common among those born outside Australia (21.9% vs 28.9%, *X*^2^ (1, *N* = 3,840) = 20.3, *p* < .001), while “health professional or someone else ACP” was more common among those born outside Australia (46.4% vs 34.8%, *X*^2^ (1, *N* = 3,840) = 45.5, *p* < .001). Strongest associations were found for those born in Southern Europe: “person completed ACD” (odds ratio [OR] = 0.56, 95% confidence interval [CI] = 0.36–0.88), and “health professional or someone else ACP” (OR = 1.41, 95% CI = 1.01–1.98). English-language proficiency and increased age significantly predicted both ACP outcomes.

**Discussion:**

Region of birth is associated with the rate and type of ACP uptake for some older Australians. Approaches to ACP should facilitate access to interpreters and be sensitive to diverse preferences for individual and family involvement in ACP.

Since European colonization in the late 18th century, Australia has experienced significant population growth through overseas migration. In 2018, 29.4% (over 7.3 million) of Australia’s population were born overseas (Australian Bureau of Statistics, 2019), giving Australia one of the highest foreign-born populations among the Organization for Economic Co-operation and Development (OECD) member states in proportionate terms (Organization for Economic Co-operation and Development, 2018). Australia’s foreign-born population is today one of the more culturally diverse internationally (Australian Multicultural Council, 2013), with a significant and growing population of older people from culturally and linguistically diverse (CaLD) backgrounds. In 2016, 37% of Australians 65 years and older were born overseas, with over half of these (18% of all older Australians) speaking a language other than English at home (Australian Bureau of Statistics, 2017). This intersection of cultural and linguistic diversity with population aging is an important consideration in health care service design, including for those with life-limiting illnesses (Johnstone et al., 2016).

A key component of delivering health and care services to a diverse population of older adults involves understanding these diverse needs and aligning care with the person’s values and preferences. Advance care planning (ACP) is a process that supports adults at any age or stage of health in understanding and sharing their personal values, life goals, and preferences regarding future medical care (Sudore et al., 2017). The ACP process may include discussions about the person’s values and preferences, completion of an instructional advance care directive (ACD) documenting specific preferences for future care or treatments, or an ACD appointing a substitute decision maker for health care. Most Australian jurisdictions have statutory legal frameworks for instructional ACDs and all jurisdictions provide for ACDs to appoint substitute decision makers for health care (Fountain et al., 2018; White et al., 2018). Where this legislation is not in place, nonstatutory ACDs may still have support under the common law. In the Australian context, while ACDs specifically refer to documents developed by the person with decision-making capacity, the term “advance care planning” has been used more broadly (Australian Health Minister’s Advisory Council, 2011). This usage acknowledges that ACP documents are sometimes completed on a person’s behalf by family members or health professionals, such as in cases where the person is incapacitated and unable to express their own wishes (Blake et al., 2017). While this approach is often motivated by pragmatic concerns to ensure high-quality end-of-life care, such documents are not considered legally binding in Australia (Australian Health Minister’s Advisory Council, 2011).

In health care systems where ACP is commonplace and endorsed, it is typically associated with practices including open and frank disclosure of medical diagnoses (“truth-telling”), shared decision making between patients and health care professionals, and an emphasis on the preferences of the person receiving care (Blackhall et al., 1995; Gysels et al., 2012; Meñaca et al., 2012). Some have suggested that the normative ideological views underpinning support for ACP across many “Western” societies are derived from an individualistic approach which prioritizes individual agency within an “autonomy-control” narrative (Gordon & Paci, 1997; Johnstone & Kanitsaki, 2009). This is contrasted with the normative practices of other societies, in which a “social-embeddedness” narrative is proposed to explain the strong involvement of nuclear and extended family, and a tendency to view open disclosure of medical diagnoses and individual choice as burdensome, rather than empowering (Gordon & Paci, 1997; Sinclair et al., 2014).

Values and beliefs associated with the “social-embeddedness” narrative are thought to be prominent within areas of Southern Europe (Gordon & Paci, 1997; Meñaca et al., 2012) and Asia (Shin et al., 2016; Zhu et al., 2020). Cross-national studies have shown higher prevalence of doctor–patient discussions about future treatment preferences and higher rates of formal appointments of substitute decision makers in Northern European (The Netherlands, Belgium) compared to Southern European (Italy, Spain) countries (Evans et al., 2013). While a systematic review of end-of-life practices across Spain, Italy, and Portugal warned against simplistic explanations based on “Southern European culture,” reliable differences were found in comparison to Northern and Western Europe with respect to partial disclosure of medical information. These differences were explained with reference to strong family involvement in care, family-based decision making and Catholic attitudes relating to gradual truth-telling (Meñaca et al., 2012). There is an established literature on collusion between family members and health care professionals to limit disclosure of medical information, typically aimed at reducing patient anxiety and often associated with cultures in which family-based decision making is more prominent (Chaturvedi et al., 2009; Gordon & Paci, 1997). Such differences may explain different trajectories of political debate and legislation to support ACP globally, and the more liberal provisions to enable self-determination adopted by some countries (Veshi & Neitzke, 2015). This diversity in observed practices suggests that cultural factors may contribute to the endorsement of ACP as a normative practice.

Even in societies in which liberal values of “individual autonomy” are strongly endorsed, there is variation in attitudes and practices associated with ACP. Within the United States, the first country to establish national legislation and policies for ACP, migrant and ethnic minority groups tend to engage in ACP at lower rates than White Americans (Blackhall et al., 1995; Johnson et al., 2008; Matsumura et al., 2002). This may reflect a pluralism of values and beliefs within a multicultural society, including the influence of cultural values from countries of origin (Bito et al., 2007). Importantly however, these ethnic disparities in ACP uptake are sensitive to sociodemographic and psychosocial factors, including education level and personal control (Inoue, 2016), socioeconomic status (Koss & Baker, 2018), health literacy (Nouri et al., 2019), religiosity, and trust in health care providers (Johnson et al., 2008). The disparity between White American and African American people in terms of ACP uptake has also reduced in recent years (Koss & Baker, 2017). The sensitivity of these disparities in ACP uptake to sociodemographic variables (which vary across ethnic groups), and their fluidity over time, means that differences in ACP uptake between ethnic groups should not be automatically attributed to cultural factors.

These findings suggest that in multicultural countries like Australia, ACP attitudes and uptake may vary across CaLD communities. While cultural factors have been described as a barrier to ACP uptake (Boddy et al., 2013; Zivkovic, 2018), a study of older Chinese Australians identified language difficulties and low levels of ACP awareness as the most important factors, concluding that the majority of older Chinese Australians saw ACP as a pragmatic response to serious illness and a way of reducing suffering among family members (Yap et al., 2018). A cross-sectional survey of older adults found that in comparison to those from “Anglo-Australian” backgrounds, those from “Mediterranean” backgrounds reported lower rates of discussing wishes about future medical treatments with family members or their general practitioner. However, these findings are complicated by the lower level of education, and higher levels of self-reported religiosity among the Mediterranean participants (Ohr et al., 2017). While these findings are informative with respect to attitudes toward ACP among CaLD community members, currently very little is known about actual ACP uptake among these groups in the Australian setting. Differences in methodological approaches, diverse migration histories and local differences in legal frameworks and service provision in the existing Australian studies make it difficult to identify and disentangle “cultural” factors from other intersecting variables. There is a need for research describing the actual use of ACP among CaLD populations, with sufficient scale and sensitivity to control for potential confounding variables (Inoue, 2016). The current study addresses this gap and explores the influence of birth country and sociodemographic variables on ACP uptake, through a national multicenter audit of ACP documentation.

## Method

This study was conducted by Advance Care Planning Australia (ACPA) as part of the *National ACD Prevalence Study*, which investigates the prevalence of ACDs and other ACP documentation in the health records of people aged 65 years or older accessing general practice (GP) clinics, hospitals, and long-term care facilities (LTCFs) in Australia.

### Study Design and Procedures

The study design was a prospective, multicenter, cross-sectional audit (Detering et al., 2019). Participating study sites (*N* = 100) were recruited through an expression of interest process, which also collected information about the site (e.g., location, jurisdiction, funding type, service size). Each site nominated one or more data collectors, who undertook compulsory online training and received a data collection manual, with information about the ACP documents relevant in each jurisdiction. Data collection occurred during defined periods (1–3 consecutive days). Participating sites nominated how many records they would audit (minimum 30, maximum 50). Health records were randomly selected on the basis of all people meeting the eligibility criteria on the first day of the defined study period. To be eligible, the person had to be admitted to the participating hospital or LTCF for at least 48 hr prior to the audit, or attending the GP clinic on the nominated study day(s). The random selection of health records from the eligible list was undertaken by the ACPA research team (www.randomizer.org) to produce a list of selected health records equivalent to the nominated number of records to be audited (i.e., 30–50) along with a supplementary list of 10 records to be used (consecutively) in case any of the selected health records were unavailable. For GP settings, consecutive eligible records were audited until the nominated target number was reached. Data collectors obtained paper and/or electronic records, including the national personally controlled electronic “My Health Record” (Australian Digital Health Agency, 2020) if applicable and attempted to locate relevant ACDs or other ACP documentation for a period of 15 min from opening the record. Information about any ACDs or ACP documentation was extracted (e.g., type of document, contents, and time taken to find), along with demographic and clinical information about the person.

### Person-Level Variables

This data set included a “country of birth” indicator coded as “born in Australia,” “born outside Australia,” or “unknown.” For those born outside Australia an additional text field captured country of birth. Where responses listed two or more countries, the first-listed country was used. After preliminary cleaning, birth countries were grouped into broader “birth regions” with reference to the United Nations classification system of Geographic Regions (United Nations Department of Economic and Social Affairs (Statistics Division), 2020). Descriptive statistics are reported using the midlevel (intracontinental) regional system; however, in cases where there were less than 30 observations for an intracontinental region, these were collapsed into continental categories for reporting and analysis (see Table 1).

**Table 1.** Sample Characteristics by Health Sector

	Sector: *N* (%)				
Variable	General practice (*n* = 676)	Hospital (*n* = 1,122)	LTCF (*n* = 2,389)	Overall (*N* = 4,187)	*p* Value
Age group					*p* < .001
65–79	499 (73.8%)	597 (53.2%)	548 (23.9%)	1,644 (39.3%)	
80+	177 (26.2%)	525 (46.8%)	1,841 (77.1%)	2,543 (60.7%)	
Gender					*p* < .001
Male	302 (44.7%)	584 (52.0%)	761 (31.9%)	1,647 (39.3%)	
Female	372 (55.0%)	535 (47.7%)	1,618 (67.7%)	2,525 (60.3%)	
Other or unknown	2 (0.3%)	3 (0.3%)	10 (0.4%)	15 (0.4%)	
Rurality/remoteness					*p* < .001
Major cities	281 (41.6%)	640 (57.0%)	1,497 (62.7%)	2,418 (57.8%)	
Regional	352 (52.1%)	461 (41.1%)	840 (35.2%)	1,653 (39.5%)	
Remote	5 (0.7%)	18 (1.6%)	50 (2.1%)	73 (1.7%)	
Unknown	38 (5.6%)	3 (0.3%)	2 (0.1%)	43 (1.0%)	
Functional status (disability level)					*p* < .001
Some or less	512 (75.7%)	285 (25.4%)	94 (3.9%)	891 (21.3%)	
Moderate or more	77 (11.4%)	734 (65.4%)	2,285 (95.6%)	3,096 (73.9%)	
Unknown	87 (12.9%)	103 (9.2%)	10 (0.4%)	200 (4.8%)	
Palliative care referral status					*p* < .001
Referred	5 (0.7%)	125 (11.1%)	197 (8.2%)	327 (7.8%)	
Not referred	425 (62.9%)	957 (85.3%)	2,179 (91.2%)	3,561 (85.0%)	
Unknown	246 (36.4%)	40 (3.6%)	13 (0.5%)	299 (7.1%)	
Morbidity level					*p* < .001
No current conditions	58 (8.6%)	13 (1.2%)	2 (0.1%)	73 (1.7%)	
Uni-morbid	177 (26.2%)	325 (29.0%)	106 (4.4%)	608 (14.5%)	
Comorbid	188 (27.8%)	235 (20.9%)	343 (14.4%)	766 (18.3%)	
Multimorbid	253 (37.4%)	549 (48.9%)	1,938 (81.1%)	2,740 (65.4%)	
Relationship status					*p* < .001
Married/de facto	335 (49.6%)	585 (52.1%)	532 (22.3%)	1,452 (34.7%)	
Divorced/separated	47 (7.0%)	102 (9.1%)	238 (10.0%)	387 (9.2%)	
Widowed	102 (15.1%)	312 (27.8%)	1,198 (50.1%)	1,612 (38.5%)	
Single	44 (6.5%)	100 (8.9%)	264 (11.1%)	408 (9.7%)	
Unknown	148 (21.9%)	23 (2.0%)	157 (6.6%)	328 (7.8%)	
Birth region					
Oceania (incl. Australia)	341 (50.4%)	731 (65.2%)	1,679 (70.3%)	2,751 (65.7%)	
Africa	3 (0.4%)	15 (1.3%)	23 (1.0%)	41 (1.0%)	
Americas	2 (0.3%)	14 (1.2%)	14 (0.6%)	30 (0.7%)	
Asia	9 (1.3%)	74 (6.6%)	89 (3.7%)	172 (4.1%)	
Eastern Europe	1 (0.1%)	17 (1.5%)	34 (1.4%)	52 (1.2%)	
Northern Europe	41 (6.1%)	107 (9.5%)	204 (8.5%)	352 (8.4%)	
Southern Europe	14 (2.1%)	113 (10.1%)	218 (9.1%)	345 (8.2%)	
Western Europe	5 (0.7%)	29 (29.9%)	63 (2.6%)	97 (2.3%)	
Unknown	260 (38.5%)	22 (2.6%)	65 (2.7%)	347 (8.3%)	
Language status					*p* < .001
Speaks English	576 (85.2%)	1,004 (89.5%)	2,170 (90.8%)	3,750 (89.6%)	
Interpreter required	1 (0.1%)	92 (8.2%)	184 (7.7%)	277 (6.6%)	
Unknown	99 (14.6%)	26 (2.3%)	35 (1.5%)	160 (3.8%)	
Religion					*p* < .001
No religion	1 (0.1%)	92 (8.2%)	90 (3.8%)	183 (4.4%)	
Christian	10 (1.5%)	706 (62.9%)	1,433 (60.0%)	2,149 (51.3%)	
Other religion	0	54 (4.8%)	70 (2.9%)	124 (3.0%)	
Unknown	665 (98.4%)	270 (24.1%)	796 (33.3%)	1,731 (41.3%)	

*Notes:* Column percentages indicate the proportionate prevalence of each variable level within each health sector. LTCF = long-term care facility. Percentages are not reported for cells with zero counts. *p* Values refer to the statistical significance of chi-squared tests for each variable (other than birth region for which small cell counts impeded analysis) across the sector categories.

A range of additional sociodemographic variables were extracted from the person’s medical record including age, gender (“Male,” “Female,” “Other,” “Unknown”), postcode, language, religion, and relationship status (“Married/de facto,” “Divorced/separated,” “Widowed,” “Single,” “Unknown”). Remoteness was classified by entering the postcode from the person’s normal place of residence into a national system for organizing remote health care resource allocation (Department of Health, 2020). Those who were identified as requiring an interpreter had their preferred (non-English) language extracted from the medical record. Documented religious beliefs were extracted if available and categorized into higher-level groups (“Buddhism,” “Christian,” “Hindu,” “Islam,” “Jewish,” “No religion,” “Other,” “Unknown”).

Clinical information collected included current/active medical conditions (categorized by organ system), and palliative care status. Functional status was classified according to the Eastern Cooperative Oncology Group (ECOG) classification system (Buccheri et al., 1996), which was extracted from the notes where available, or else estimated by the data collector based on other information in the notes (Detering et al., 2019).

### ACP Documentation

The types of ACP documentation extracted from the medical records were classified according to whether they were completed by the person concerned, by a health professional, or by someone else (e.g., family member or substitute decision maker). Documents completed by the person included ACDs (e.g., an instructional ACD or an ACD appointing a substitute decision maker) or other forms of ACP completed by the person. Documents completed by health professionals included medical orders, clinical care plans, and/or other ACP documentation (e.g., record of an ACP discussion). Documents completed by someone else included any ACP documentation by the person’s family, substitute decision maker(s), or other person. The primary outcome measures were the prevalence of cases in which the person completed any type of ACD (“person completed ACD”), and cases in which a health professional or someone else completed some form of ACP documentation on the person’s behalf, in the absence of a person completed ACD (“health professional or someone else ACP”). The “person completed ACD” variable is thought to reflect the presence of ACP in which the individual was significantly involved in the process, while the “health professional or someone else ACP” variable is taken as a proxy indicator of cases in which the individual had less or no involvement in the ACP. While this variable may to some extent reflect documentation of family-based ACP discussions, or unilateral clinical decision making due to the person’s impaired decision-making capacity, it is also sensitive to cases in which the person deferred or delegated decision making to others, or where limited disclosure (e.g., by family members or health professionals) prevented their involvement in decision making. As such these two variables may be considered as proxy indicators for practices associated with the “autonomy-control” and “social-embeddedness” narratives described earlier (Gordon & Paci, 1997).

### Data Analysis

Variable re-coding and data analyses were conducted in R Studio (version 1.2.1335). Initial descriptive analyses tabulated prevalence rates by birth region and health sector (GP, hospital, and LTCF). Sample representativeness was assessed by comparing birth region prevalence to population census data. Bivariate relationships between sample characteristic variables were explored across health sector with chi-squared tests. The primary research questions were tested by fitting separate hierarchical multilevel logistic regression models for the two outcome variables (“person completed ACD” and “health professional or someone else ACP”) with study site included as a random effect, to allow for clustering of data within sites. This approach was justified by preliminary exploratory analyses, which indicated that rates of ACP documentation by site ranged from 0% to 100%, and intraclass correlation coefficients indicating 35–43% of variance accounted for by “between-site” factors. Generalized linear models were fitted using the *glmer* function from the *lme4* package with Laplace estimation and allowing 20,000 iterations for model convergence. Models were fitted to a subset of the data (*n* = 3,619) containing only the four most populous birth regions: Oceania and Australia (*n* = 2,751), Northern Europe (*n* = 352), Southern Europe (*n* = 345), and Asia (*n* = 172). Individual-level variables were re-scaled (age was mean-centered) and categorical variables were collapsed as follows to increase model parsimony: gender (female [reference]; male; other or unknown), remoteness (major cities [reference]; regional; remote; unknown), relationship status (currently partnered [reference]; previously partnered; other or unknown), English-language status (speaks English or unknown [reference]; interpreter required), religion (no religion [reference]; Christian; all other religions; unknown), functional status (some disability or less [reference]; moderate disability or more; unknown), morbidity (no current conditions [reference]; one or more conditions), and palliative care referral status (not referred or unknown [reference]; referred).

Initial empty (intercept only) and single-predictor (intercept and study site) models were generated to justify the multilevel approach, through model chi-squared comparisons and intraclass correlation coefficients. Model fitting proceeded hierarchically, with addition of birth region (Model 1), sociodemographic individual-level variables (Model 2), and finally clinical individual-level variables and two-way interaction terms (Model 3). Model fit was assessed at each stage using deviance statistics (−2 log likelihood and Akaike’s Information Criterion).

## Results

In total, 4,187 health records were audited, from 100 participating sites across all eight Australian jurisdictions. The characteristics of the sample across the three health sectors (GP, hospital, and LTCF) are shown in Table 1 (see also Supplementary File 1 for sample characteristics by region of birth). Of the 3,839 audited records in which country of birth was known and reported, 1,152 (30.0%) participants were born outside Australia, comparable with population census statistics (29.4%, *z* = 0.82, *ns*). When considering the proportions from the continental and intracontinental regions reported in Table 1, participants from Western Europe (2.53% vs 1.05%, *z* = 9.0, *p* < .001), Northern Europe (9.17% vs 5.10%, *z* = 11.5, *p* < .001), Southern Europe (8.99% vs 2.19%, *z* = 28.7, *p* < .001), and Eastern Europe (1.35% vs 0.63%, *z* = 5.7, *p* < .001) were more prevalent in this sample than in the broader Australian population, while those from Asia (4.45% vs 13.4%, *z* = −16.3, *p* < .001), Americas (0.78% vs 1.38%, *z* = −3.16, *p* < .001), and Africa (1.07% vs 1.83%, *z* = 3.52, *p* < .001) had a lower prevalence than in the Australian population (Australian Bureau of Statistics, 2019).

The rates of ACP documentation are shown in Table 2. Across the entire sample, the prevalence of cases in which an ACD was completed by the person (“person completed ACD”) was 25.3% (1,061/4,187). The prevalence of cases in which a health professional or other person completed some ACP documentation on behalf of the person was 48.4% (2,026/4,187), and the subset of cases in which this occurred in the absence of any ACD completed by the person (hence a “health professional or someone else ACP”) was 36.6% (1,532/4,187). The prevalence of the “person completed ACD” variable was lower among those born outside Australia (21.9%) compared to those born in Australia (28.9%), (*X*^2^ (1, *N* = 3,840) = 20.3, *p* < .001). However the prevalence of the “health professional or someone else ACP” variable was higher among those born outside Australia (46.4%) compared to those born in Australia (34.8%), (*X*^2^ (1, *N* = 3,840) = 45.5, *p* < .001).

**Table 2.** ACP Documentation of Different Types (Completed by the Person, Health Professional, and/or Someone Else) by Birth Region and Health Sector (Unweighted Prevalence Rates)

Type of ACP documentation and person who completed: *N* (%)						
Variable	“Person completed ACD” ^a^	Health professional ACP documentation	Someone else ACP documentation	“Health professional or someone else ACP” ^b^	Any type ACP	Total
Birth region						
Oceania and Australia*	793 (28.8%)	936 (34.0%)	501 (18.2%)	958 (34.8%)	1,751 (63.6%)	2,751
Africa	5 (12.2%)	14 (34.1%)	12 (29.3%)	19 (46.3%)	24 (58.5%)	41
Americas	4 (13.3%)	13 (43.3%)	4 (13.3%)	14 (46.7%)	18 (60.0%)	30
Asia	38 (22.1%)	71 (41.3%)	47 (27.3%)	77 (44.8%)	115 (66.9%)	172
Eastern Europe	8 (15.4%)	26 (50.0%)	14 (26.9%)	27 (51.9%)	35 (67.3%)	52
Northern Europe	104 (29.5%)	141 (40.1%)	56 (15.9%)	133 (37.8%)	237 (67.3%)	352
Southern Europe	45 (13.0%)	196 (56.8%)	82 (23.8%)	203 (58.8%)	248 (71.9%)	345
Western Europe	32 (33.0%)	44 (45.4%)	17 (17.5%)	38 (39.2%)	70 (72.2%)	97
Other or unknown	32 (9.2%)	64 (18.4%)	24 (6.9%)	63 (18.1%)	95 (27.4%)	347
Health sector						
General practice	37 (5.5%)	42 (6.2%)	2 (0.3%)	37 (5.5%)	44 (6.5%)	676
Hospital	124 (11.1%)	607 (54.1%)	29 (2.6%)	516 (46.0%)	615 (54.8%)	1,122
Long-term care (LTC)	900 (37.7%)	856 (35.8%)	726 (30.4%)	979 (41.0%)	1,367 (57.2%)	2,389
Total	1,061 (25.3%)	1,505 (35.9%)	757 (18.1%)	1,532 (36.6%)	2,026 (48.4%)	4,187

*Notes:* A health record may contain more than one type of advance care planning (ACP) documentation; hence, columns are not additive. *The “Oceania (incl. Australia)” birth region included *n* = 2,688 people born in Australia, *n* = 48 born in New Zealand, and *n* = 15 born in Papua New Guinea or Pacific Islands.

^a^This column counts statutory and nonstatutory advance care directives (ACDs) completed by the person and equates to the “Person completed ACD” outcome variable.

^b^This column equates to the “Health professional or someone else ACP” outcome variable (which is counted only for cases in which there is no ACD completed by the person themselves).

The first logistic regression model used the “person completed ACD” outcome variable. The intraclass correlation coefficient from the single-predictor variable model (study site as a random effect) was 0.43, indicating that 43% of variance was explained by “study site” level factors. The stages in the hierarchical model-building process are shown in Table 3. Model 1 indicated a lower likelihood of “person completed ACD” among those born in the Southern European region (odds ratio [OR] = 0.49, 95% confidence interval [CI] = 0.33–0.75). Model 2 showed that this effect remained significant following inclusion of other sociodemographic variables. Significant main effects included an increased likelihood of “person completed ACD” with each increasing year of age (OR = 1.03, 95% CI = 1.02–1.05), reduced likelihood among males (OR = 0.80, 95% CI = 0.66–0.97), and reduced likelihood among those requiring an interpreter (OR = 0.54, 95% CI = 0.29–0.99). Model 3 showed that the effect of birth region and sociodemographic variables remained significant with the inclusion of clinical variables. There was a trend toward an increased likelihood of “person completed ACD” among those receiving palliative care services, although this fell short of statistical significance. Functional status and morbidity were not predictive and are not reported in the final Model 3. The inclusion of significant two-way interactions showed that the effect of increasing age on the likelihood of “person completed ACD” was stronger for males (OR = 1.03, 95% CI = 1.01–1.06). Nagelkerke’s pseudo-*R*-squared for the final Model 3 was 0.38.

**Table 3.** Model Statistics for Predictors of an ACD Being Completed by the Person (“Person Completed ACD”)

Predictors	Odds	95% CI	Odds	95% CI	Odds	95% CI
Model 1						
Birth region (Northern Europe)	1.11	0.83–1.48				
Birth region (Southern Europe)	0.49**	**0.33–0.75**				
Birth region (Asia)	0.79	0.45–1.36				
Model 2						
Birth region (Northern Europe)			1.08	0.81–1.45		
Birth region (Southern Europe)			0.56*	**0.36–0.87**		
Birth region (Asia)			0.90	0.51–1.60		
Age (years)			1.03***	**1.02–1.05**		
Gender (male)			0.80*	**0.66–0.97**		
Gender (other/unknown)			1.52	0.45–5.15		
English-speaking status (interpreter required)			0.54*	**0.29–0.99**		
Model 3						
Birth region (Northern Europe)					1.07	0.80–1.43
Birth region (Southern Europe)					0.56*	**0.36–0.88**
Birth region (Asia)					0.90	0.51–1.60
Age (years)					1.02***	**1.01–1.04**
Gender (male)					0.77**	**0.64–0.93**
Gender (other/unknown)					1.52	0.44–5.31
English-speaking status (interpreter required)					0.52*	**0.28–0.96**
Palliative care referral status (yes)					1.40	0.96–2.02
Age * Gender (male)					1.03**	**1.01–1.06**
Age * Gender (other/unknown)					1.04	0.91–1.21

*Notes:* ACD = advance care directive; CI = confidence interval. Model 1 includes birth region. Model 2 includes additional sociodemographic variables. Model 3 includes additional clinical variables and two-way interactions. For birth region variable the reference category is “Oceania and Australia.” For gender variable the reference category is “Female.” Predictors and coefficients with statistically significant effects (*p* < .05) are listed in boldface.

*p < .05. **p < .01. ***p < .001.

The second logistic regression model used the “health professional or someone else ACP” variable. The intraclass correlation coefficient was 0.35, indicating that 35% of variance was explained by “study site” level factors. The stages in the hierarchical model-building process are shown in Table 4. Model 1 indicated a higher likelihood of having some form of ACP documentation completed by a health professional or other person among those born in the Southern European region (OR = 1.65, 95% CI = 1.21–2.25). In Model 2 this effect was attenuated with the inclusion of other sociodemographic variables, falling below the level of statistical significance (OR = 1.36, 95% CI = 0.97–1.90). Significant main effects included an increased likelihood of “health professional or someone else ACP” with each increasing year of age (OR = 1.02, 95% CI = 1.01–1.03), and increased likelihood among those requiring an interpreter (OR = 1.73, 95% CI = 1.14–2.62). Remoteness, relationship status, religious group, and gender were not predictive and were removed. Model 3 showed that the effect of birth region and sociodemographic variables remained significant after the inclusion of clinical variables. Those with poorer functional status (moderate disability or more, OR = 2.11, 95% CI = 1.59–2.80) and those receiving palliative care (OR = 2.99, 95% CI = 2.17–4.12) had increased likelihood of “health professional or someone else ACP.” There were no significant two-way interactions. Nagelkerke’s pseudo-*R*-squared for the final Model 3 was 0.34.

**Table 4.** Model Statistics for Predictors of Some Form of ACP Being Completed by a Health Professional or Other Person, in the Absence of an ACD Completed by the Person (“Health Professional or Other ACP”)

Predictors	Odds	95% CI	Odds	95% CI	Odds	95% CI
Model 1						
Birth region (Northern Europe)	1.19	0.91–1.55				
Birth region (Southern Europe)	1.65**	**1.21–2.25**				
Birth region (Asia)	0.84	0.55–1.27				
Model 2						
Birth region (Northern Europe)			1.18	0.90–1.53		
Birth region (Southern Europe)			1.36	0.97–1.90		
Birth region (Asia)			0.71	0.45–1.11		
Age (years)			1.02***	**1.01–1.03**		
English-speaking status (interpreter required)			1.73**	**1.14–2.62**		
Model 3						
Birth region (Northern Europe)					1.15	0.88–1.51
Birth region (Southern Europe)					1.41*	**1.01–1.98**
Birth region (Asia)					0.73	0.47–1.16
Age (years)					1.01**	**1.00–1.03**
English-speaking status (interpreter required)					1.67*	**1.10–2.53**
Functional status (moderate disability or more)					2.11***	**1.59–2.80**
Functional status (unknown)					1.01	0.50–2.00
Palliative care referral status (yes)					2.99***	**2.17–4.12**

*Notes:* ACD = advance care directive; ACP = advance care planning; CI = confidence interval. Model 1 includes birth region. Model 2 includes additional sociodemographic variables. Model 3 includes additional clinical variables and two-way interactions. For birth region variable the reference category is “Oceania and Australia.” For functional status variable the reference category is “Some disability or less.” Predictors and coefficients with statistically significant effects (*p* < .05) are listed in boldface.

*p < .05. **p < .01. ***p < .001.

## Discussion

This prospective, multicenter, cross-sectional audit study investigated associations between country of birth and different forms of ACP in the health records of older people accessing GP clinics, hospitals, and LTCFs in Australia. A higher prevalence of “person completed ACDs,” and lower prevalence of “health professional or someone else ACP,” was observed for those born in Australia compared to those born outside Australia, with strong effects for those born in Southern Europe in multivariate models.

At a broad level, just over a quarter of the audited health records contained an ACD completed by the person. Approximately one third (36.6%) of records were counted as cases in which a health professional or someone else completed some form of ACP documentation on the person’s behalf, in the absence of any documentation by the person themselves. This variable is interpreted as a proxy indicator for patterns of health care decision making associated with the “social-embeddedness” narrative (Gordon & Paci, 1997), which have been proposed to be more prominent in parts of Asia and Southern Europe. The study partially upheld this hypothesis, with multivariate models showing that those born in the Southern European region had a lower likelihood of completing their own ACD, and a higher likelihood of having ACP documents completed on their behalf by health professionals or other people, when compared to those born in the Oceania and Australia region. This effect remained significant even when controlling for English-language proficiency (which was also a significant independent predictor of both outcome variables) and religious group (which was not a significant predictor). These findings are consistent with previous Australian studies (Ohr et al., 2017; Sinclair et al., 2014) and cross-national European studies (Evans et al., 2013; Meñaca et al., 2012). One interpretation is that cultural values associated with certain countries of birth may influence communication styles between patients, health professionals, and family members. Although a similar pattern of results was evident in group means for those born in Asian countries, this was not significant in the regression models. This may reflect that normative health care decision-making practices in this group are more similar to those born in the Oceania and Australia region. Alternatively, the lack of a significant effect could indicate diversity in practices among those from countries grouped within the “Asia” geographic region. Similar trends were observed for those from Eastern Europe and Africa, although small sample sizes precluded multivariate analyses. The international literature is sparse regarding ACP uptake among these groups. Given contemporary migration patterns in Australia, particularly from Asian and African countries, further research should investigate ACP among these groups.

Increasing age was an independent predictor of ACP documentation across all groups, for both the “person completed ACD” and “health professional or someone else ACP” outcome variables. These findings are consistent with previous studies (Sellars et al., 2020), suggesting that increasing age exposes people to multiple factors associated with increased ACP uptake, including declining health, increased contact with health care systems, and experiences with end-of-life care among family and friends (Amjad et al., 2014). The observation of a lower likelihood of “person completed ACDs” among males compared to females adds to the mixed pattern of findings regarding the association between sex and ACP uptake (Carr & Khodyakov, 2007; Rurup et al., 2006; White et al., 2019).

Those who required an interpreter had a lower likelihood of completing their own ACD, and a higher likelihood of having ACP documents completed for them by a health professional or other person. Those who speak English proficiently may be more able to engage directly with ACD forms and the broader health care system to make their preferences known. English-language proficiency may also be related to ACP uptake via other variables such as health literacy (de Vries et al., 2019). Alternatively, among a migrant group within a predominantly English-speaking country, English-language proficiency may indicate the person’s level of “acculturation” into the host nation, which may be accompanied by adoption of cultural values consistent with a positive attitude toward ACP (Bito et al., 2007). Facilitated ACP interventions addressing linguistic diversity (e.g., with assistance from interpreters trained in ACP) have been shown to be feasible in a hospital setting (Detering et al., 2015). This suggests that targeted interventions may be effective in promoting ACP uptake within some CaLD populations in which a lack of English-language proficiency is a barrier.

Those with poorer functional status and those receiving palliative care were more likely to have “health professional or someone else ACP.” While palliative care status showed a trend toward an increased likelihood of “person completed ACD,” it appears that clinical variables were more influential on the processes of ACP or clinical decision making that occurred without the involvement of the person themselves, perhaps in response to acute clinical deterioration, judgments of “clinical futility,” or planning in the context of a person with already impaired decision-making capacity. However, the fact that the main hypothesized effects remained significant even while controlling for these clinical variables suggests that clinical decision-making processes were not driving the effects of birth region or English-language proficiency. Future research might explore specific disease types and trajectories in which the person’s involvement in completing their own ACD is compromised (e.g., advanced dementia).

### Limitations

This study has a number of limitations, which should be considered in the interpretation of the results. The cross-sectional, observational design means that causality cannot be inferred. Although significant measures were taken to maximize sample representativeness (e.g., geographically diverse study sites) the eligibility criteria for inclusion (65 years or older and currently accessing the health setting) meant the study sample was older and more functionally impaired than the underlying population. There was a higher prevalence of people born in Europe, and a lower prevalence of people from Africa, Asia, or Americas, than would be expected based on population census data (Australian Bureau of Statistics, 2019). While the differences in prevalence of “person completed ACDs” between those born within versus outside Australia (28.9% vs 21.8%) were not dramatic, the relative prevalence of different birth regions within the sample, each with different rates of ACP uptake, illustrates the importance of considering region-specific adjusted model estimates alongside the raw prevalence data. The analysis was limited by a lack of data on the time of migration to Australia among the foreign-born population. Without this information it is difficult to determine whether the observed effects of birth region do persist among those who have spent significant periods of time in Australia. There were higher rates of “unknown” values recorded for sociodemographic variables (e.g., country of birth and English-speaking status) in the GP setting. This may reflect the brief and episodic nature of primary care interactions and the nature of the health records kept for these patients. Finally, the current study was unable to control for other relevant sociodemographic characteristics such as education level or socioeconomic status, both of which have been shown to influence engagement with ACP.

### Implications

The current study indicates that certain CaLD communities may have enduring differences in normative practices relating to health care decision making, with implications for ACP implementation and uptake. The sociodemographic and clinical variables measured in this audit study attenuated these effects to some extent, and other unmeasured variables (e.g., education, socioeconomic status) may also play a role in ACP attitudes and uptake. It is important to note that ACP undertaken on a person’s behalf, by a health professional or another person (e.g., family member), may not reflect the person’s own preference. It is also important that health care services are sensitive and responsive to cultural diversity among their communities; recent resources have begun to collate information about cultural factors in end-of-life care for Australian CaLD communities (Pereira-Salgado et al., 2018). It is also important to recognize the significant diversity within CaLD communities, and to avoid cultural stereotyping in care provision. The strong, independent association between English-language proficiency and ACP uptake suggests the importance of tailoring ACP interventions and involving trained interpreters where appropriate (Detering et al., 2015). More broadly, it is important that approaches to ACP can be flexible and responsive to the preferred decision-making styles of patients and their families or chosen support networks, rather than being narrowly focused around documenting preferences on a prescribed instructional ACD form (Zivkovic, 2018). Respect for autonomy includes respecting a person’s cultural worldview and the values that inform their preferred decision-making style.

## Conclusion

The current study is the first to report rates of ACP documentation in the health records of people from CaLD community groups in Australia, and attempt to understand these with reference to relevant sociodemographic variables. This study suggests that some CaLD community groups may tend toward a “social-embeddedness” rather than an “autonomy-control” approach to health care decision making. This challenges the assumptions embedded in an “individualist” approach to ACP, particularly the emphasis on instructional ACDs as the main approach. To truly respect autonomy and reflect personal values and preferences, ACP processes should be flexible and accommodate diversity in preferred decision-making styles.

## Funding

This work was supported by the Australian Government Department of Health.

## Conflict of Interest

None declared.

## Supplementary Material

gbaa127_suppl_Supplementary_Material
