# Supplementary material for: Association Between Region of Birth and Advance Care Planning Documentation Among Older Australian Migrant Communities: A Multicenter Audit Study
Source: J Gerontol B Psychol Sci Soc Sci. 2020 Aug 17;76(1):109–20. doi: 10.1093/geronb/gbaa127 (PMC7756686; doi:10.1093/geronb/gbaa127)
Supplement: gbaa127_suppl_Supplementary_Material [file gbaa127_suppl_supplementary_material.docx]

**Supplementary Table 1**

*Sample characteristics by geographic region of birth.*

___________________________________________________________________________

Variable Region of Birth: *N* (%)

___________________________________________________________________________

Oceania and Northern Southern Asia

Australia Europe Europe

(*n* = 2751) (*n* = 352) (*n* = 345) (*n* = 172)

___________________________________________________________________________

Age Group

65-79 1063 (38.6%) 126 (35.8%) 87 (25.2%) 66 (38.4%)

80+ 1688 (61.4%) 226 (64.2%) 258 (74.8%) 106 (61.6%)

___________________________________________________________________________

Gender

Male 1056 (38.4%) 132 (37.5%) 153 (44.3%) 72 (41.9%)

Female 1685 (61.3%) 218 (61.9%) 192 (55.7%) 98 (57.0%)

Other or Unknown 10 (0.4%) 2 (0.6%) 0 2 (0.4%)

___________________________________________________________________________

Rurality/Remoteness

Major Cities 1438 (52.3%) 200 (56.8%) 316 (9.2%) 146 (84.9%)

Regional 1214 (44.1%) 141 (40.1%) 28 (8.1%) 26 (15.1%)

Remote 68 (2.5%) 4 (1.1%) 0 0

Unknown 31 (1.1%) 7 (2.0%) 1 (0.3%) 0

___________________________________________________________________________

Functional Status (Disability Level)

Some or less 512 (18.6%) 66 (18.8%) 45 (13.0%) 23 (13.4%)

Moderate or more 2110 (76.7%) 270 (76.7%) 295 (85.5%) 142 (82.6%)

Unknown 129 (4.7%) 16 (4.5%) 5 (1.4%) 7 (4.1%)

___________________________________________________________________________

Palliative Care Referral Status

Referred 211 (7.7%) 32 (9.1%) 31 (9.0%) 13 (7.6%)

Not Referred 2405 (87.4%) 298 (84.7%) 302 (87.5%) 148 (86.0%)

Unknown 135 (4.9%) 22 (6.2%) 12 (3.5%) 11 (6.4%)

___________________________________________________________________________

Morbidity Level

No current conditions 36 (1.3%) 5 (1.4%) 4 (1.2%) 2 (1.2%)

Uni-morbid 403 (14.6%) 53 (15.1%) 26 (7.5%) 38 (22.1%)

Co-morbid 499 (18.1%) 64 (18.2%) 48 (13.9%) 27 (15.7%)

Multi-morbid 1813 (65.9%) 230 (65.3%) 267 (77.4%) 105 (61.0%)

___________________________________________________________________________

Relationship Status

Married / de-facto 883 (32.1%) 139 (39.5%) 133 (38.6%) 75 (43.6%)

Divorced / separated 260 (9.5%) 31 (8.8%) 35 (10.1%) 15 (8.7%)

Widowed 1087 (39.5%) 139 (39.5%) 152 (44.0%) 72 (41.9%)

Single 323 (11.7%) 22 (6.2%) 13 (3.8%) 7 (4.1%)

Unknown 198 (7.2%) 21 (6.0%) 12 (3.5%) 3 (1.7%)

___________________________________________________________________________

Variable Region of Birth: *N* (%)

___________________________________________________________________________

Oceania and Northern Southern Asia

Australia Europe Europe

(*n* = 2751) (*n* = 352) (*n* = 345) (*n* = 172)

___________________________________________________________________________

Language Status

Speaks English 2674 (97.2%) 345 (98.0%) 192 (55.7%) 78 (45.3%)

Interpreter required 12 (0.4%) 1 (0.3%) 151 (43.8%) 85 (49.4%)

Unknown 65 (2.4%) 6 (1.7%) 2 (0.6%) 9 (5.2%)

___________________________________________________________________________

Religion

No religion 136 (4.9%) 15 (4.3%) 7 (2.0%) 9 (5.2%)

Christian 1445 (52.5%) 191 (54.3%) 290 (84.1%) 73 (42.4%)

Other religion 50 (1.8%) 4 (1.1%) 9 (2.6%) 42 (24.4%)

Unknown 1120 (40.7%) 142 (40.3%) 39 (11.3%) 48 (27.9%)

___________________________________________________________________________

*Note*. Column percentages indicate the proportionate prevalence of each variable level within each geographic region of birth. Only the four most populous regions of birth are reported. Percentages are not reported for cells with zero counts.
